# Prognostic value of a novel scoring system using inflammatory response biomarkers in non‐small cell lung cancer: A retrospective study

**DOI:** 10.1111/1759-7714.13085

**Published:** 2019-05-18

**Authors:** Yan Wang, Xu Hu, Wenying Xu, Haoyuan Wang, Yu Huang, Guowei Che

**Affiliations:** ^1^ Department of Thoracic Surgery, West China Hospital Sichuan University Chengdu China; ^2^ West China School of Medicine Sichuan University Chengdu China

**Keywords:** Inflammatory response biomarker score, lymphocyte‐to‐monocyte ratio, neutrophil‐to‐lymphocyte ratio, platelet‐to‐lymphocyte ratio; non‐small cell lung cancer

## Abstract

**Background:**

The neutrophil‐to‐lymphocyte ratio (NLR), platelet‐to‐lymphocyte ratio (PLR), and lymphocyte‐to‐monocyte ratio (LMR) are reported to show a strong correlation with prognosis in patients with non‐small cell lung cancer (NSCLC). We aimed to describe a novel scoring system combining these ratios, termed the inflammatory response biomarker (IRB) score, and test its prognostic value in NSCLC.

**Methods:**

The data of 261 NSCLC patients who underwent thoracoscopic radical resection in a single center were retrospectively reviewed. The IRB score was defined as follows: a high NLR (> 2.12), a high PLR (92.9), and a low LMR (< 4.57) were each scored as 1; the opposite values were scored as 0. The individual scores were added to produce the IRB score (range: 0–3).

**Results:**

Multivariate analyses indicated that high tumor node metastasis (TNM) stage (hazard ratio [HR] 2.721, 95% confidence interval [CI] 1.597–4.989; *P* < 0.001) and an IRB score ≥ 2 (HR 2.696, 95% CI 1.506–4.826; *P* = 0.001) were independent prognostic factors for poor overall survival. Furthermore, smoking history (HR 2.953, 95% CI 1.086–8.026; *P* = 0.034), high TNM stage (HR 3.108, 95% CI 1.911–5.056; *P* < 0.001), and IRB score ≥ 2 (HR = 2.316, 95% CI: 1.389–3.861; *P* = 0.001) were demonstrated to be independent prognostic factors for poor disease‐free survival.

**Conclusion:**

The novel scoring system combining NLR, PLR, and LMR was an independent prognostic factor in NSCLC patients undergoing thoracoscopic radical resection and was superior to these ratios alone for predicting prognosis.

## Introduction

Lung cancer is the leading cause of cancer death worldwide and non‐small cell lung cancer (NSCLC) accounts for approximately 85% of all lung cancers.^1^, ^2^ Despite great advances in methods of diagnosis, treatment and follow‐up, the prognosis of lung cancer remains unsatisfactory as a result of local tumor recurrence and distant metastasis in China,^3^ the median survival duration of lung cancer patients is only 22.7 months.^4^ Therefore, efficient and reliable prognostic factors that could guide clinicians to develop the most appropriate therapeutic strategies are urgently needed.

Previous studies have revealed that age, gender, smoking, and tumor node metastasis (TNM) stage are reliable prognostic markers for lung cancer.^5^, ^6^, ^7^, ^8^ Nevertheless, patients at the same TNM stage may still have different clinical outcomes.^9^ There are also some novel biomarkers that are significantly associated with the survival of lung cancer patients and can effectively guide clinical treatments, such as EGFR and IDM‐1.^10^, ^11^ However, these markers are costly and time‐consuming to measure. Therefore, there are currently no valuable prognostic factors that can be easily obtained to precisely predict the survival of lung cancer patients.

In recent years, the systemic inflammatory response (SIR) has been proven to play a key role in cancer progression, development, and metastasis.^12^ The neutrophil‐to‐lymphocyte ratio (NLR), platelet‐to‐lymphocyte ratio (PLR), and lymphocyte‐to‐monocyte ratio (LMR) are good markers of the SIR and have shown a significant correlation with clinical outcomes in multiple tumors.^13^, ^14^, ^15^ In particular, they are simple to derive and economical. However, almost all studies on these factors only focused on a single indicator and their results were inconsistent. Therefore, we hypothesized that a scoring system combining these ratios may possess higher prognostic value than a single ratio.

In the current study, we described a novel prognostic scoring system using the NLR, PLR, and LMR that we termed the inflammatory response biomarker (IRB) score, and evaluated its prognostic significance in NSCLC patients undergoing thoracoscopic radical resection.

## Methods

### Study design

This is a retrospective study from a single center. All procedures performed involving human participants were conducted in accordance with the standards of the Ethics Committee of West China Hospital, Sichuan University and National Research Committee, and the 1964 Helsinki Declaration and its later amendments or comparable ethical standards. Because of the retrospective design of the study, informed consent was not required.

### Study population

The data of patients diagnosed with NSCLC in the same medical group at the Department of Thoracic Surgery, West China Hospital, between 1 January 2014 and 29 February 2016 were reviewed. NSCLC diagnoses were made pathologically with bronchoscopic biopsies, computed tomography‐guided needle specimens, or surgically resected specimens. The inclusion criteria were as follows: (i) pathologically confirmed NSCLC; (ii) patient underwent thoracoscopic radical lung resection and lymph node dissection; and (iii) blood tests were taken within one week preoperatively. The exclusion criteria were as follows: (i) patient received neoadjuvant therapy; (ii) clinical evidence of preoperative inflammatory condition or infection, such as inflammatory bowel disease or rheumatoid arthritis; (iii) other malignancies present; (iv) recurrent tumors; (v) patient underwent another surgery within three months before thoracoscopic radical resection; (vi) surgery was altered to thoracotomy; and (vii) insufficient data.

### Data collection

All clinicopathological data was extracted from electronic medical records, including gender, age, smoking history, preoperative comorbidity, preoperative lung function represented by forced expiratory volume in one second (FEV1), forced vital capacity (FVC) and FEV1/FVC, size, pathology, extent of resection, TNM stage (based on the 7th Union for International Cancer Control TNM classification), and laboratory data.

### Calculation and definition of neutrophil‐to‐lymphocyte ratio (NLR), platelet‐to‐lymphocyte ratio (PLR), and lymphocyte‐to‐monocyte ratio (LMR) and inflammatory response biomarker (IRB) score

The NLR was defined as a simple ratio between the absolute neutrophil and lymphocyte counts. The PLR was defined as a simple ratio between the absolute platelet and lymphocyte counts. The LMR was defined as a simple ratio between the absolute lymphocyte and monocyte counts.

The optimal NLR, PLR, and LMR cutoff values for predicting death were determined by receiver operating curve (ROC) analysis. For the NLR, the cutoff value and the area under the curve (AUC) were 2.12 and 0.672, respectively, with a sensitivity of 60.0% and a specificity of 58.7%. For the PLR, the cutoff value and the AUC were 92.9 and 0.715, respectively, with a sensitivity of 67.7% and a specificity of 53.1%. For the LMR, the cutoff value and the AUC were 4.57 and 0.679, respectively, with a sensitivity of 69.2% and a specificity of 56.6%.

The IRB score was defined as follows: a high NLR (> 2.12), a high PLR (> 92.9) and a low LMR (< 4.57) were each scored as 1; the opposite values were scored as 0. The individual scores were added to determine the IRB score (range: 0–3).

### Follow‐up and the endpoint event

Follow‐up information was obtained via telephone or directly from outpatient clinic records. The primary endpoint events of the current study were overall survival (OS) and disease‐free survival (DFS). OS was defined as the interval from the date of surgery to the date of death from any cause or the last visit, and DFS was defined as the interval from the date of surgery to the date of recurrence, metastasis, or last follow‐up. Patients known to be alive at the last follow‐up were censored.

### Statistical analysis

We used Microsoft Office Excel 2007 for data collection and SPSS version 22.0 for statistical analysis. Continuous and categorical variables were presented as median (range) values and numbers of patients (%), respectively. Student's *t*‐test or one‐way analysis of variance were used to compare continuous variables and χ2 or Fisher's exact tests for comparison of categorical variables. The OS was calculated via Kaplan–Meier analysis and the differences between groups were assessed via the log‐rank test. Prognostic factors for decreased OS rates were identified via the Cox regression model.

Univariate regression analyses were used to identify potential risk factors from the variables that seemed to be associated with prognosis based on clinical knowledge and previous studies; variables with a *P* value < 0.10 were included into multivariate regression analyses. To avoid the effect of NLR, PLR, and LMR on the IRB score in the Cox regression model, two models excluding and including the IRB score were constructed.

All statistical tests were two‐sided and *P* values < 0.05 were considered statistically significant.

## Results

### Patients characteristics

**Figure 1:**
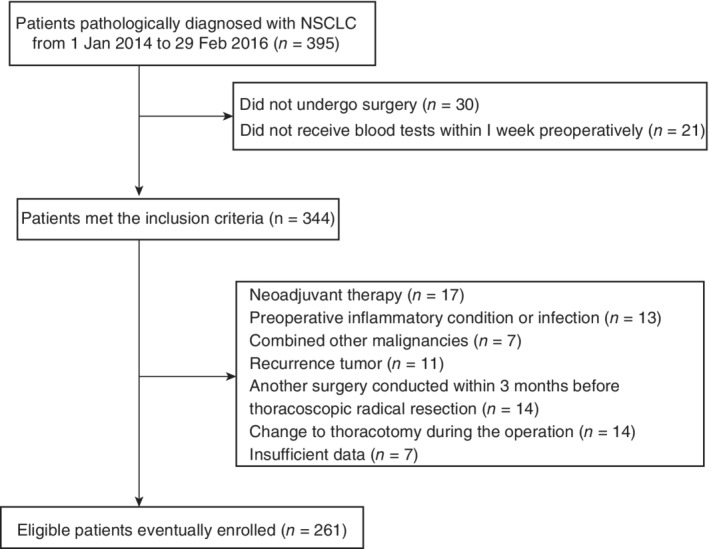
Patient selection process.

We enrolled 261 patients into our analysis based on the inclusion and exclusion criteria. The patient screening process is shown in Figure 1. The median follow‐up was 38 (range: 1–56) months. Among the 261 NSCLC patients, 144 (55.17%) were male and 123 (47.13%) were current or ex‐smokers. Regarding preoperative comorbidities, 130 (49.81%) patients had at least one kind of comorbidity, including hypertension, diabetes mellitus, chronic obstructive pulmonary disease, coronary heart disease, and emphysema. Regarding the pathological type, 190 (72.80%) cases were adenocarcinomas, which accounted for the majority of the sample population. One hundred ninety (72.80%) patients underwent lobectomy and 134 (51.34%), 80 (30.65%), and 47 (18.01%) patients were in TNM stages I, II, and III, respectively. Detailed information is shown in Table 1.

**Table 1:** Baseline clinicopathological characteristics

Characteristics	N (%)
Male	144 (55.17)
Smoking history	123 (47.13)
Preoperative comorbidity	
Hypertension	79 (30.27)
Diabetes mellitus	26 (9.96)
COPD	39 (14.94)
CHD	12 (4.6)
Emphysema	43 (16.48)
Any	130 (49.81)
Pathology	
AC	190 (72.8)
SC	46 (17.62)
Others	25 (9.58)
Extent of resection	
Lobectomy	190 (72.8)
Segmentectomy	71 (27.2)
TNM stage	
I	134 (51.34)
II	80 (30.65)
III	47 (18.01)

AC, adenocarcinoma; COPD, chronic obstructive pulmonary disease; CHD, coronary heart disease; SC, squamous carcinoma; TNM, tumor node metastasis.

### Associations between the NLR, PLR, and LMR and clinicopathologic characteristics

After grouping the 261 patients according to the optimal cutoff values of NLR, PLR and LMR, we explored the associations between these ratios and patient characteristics. The NLR was significantly correlated with gender (*P* = 0.007), age (*P* = 0.017), smoking history (*P* = 0.002), preoperative hypertension (*P* = 0.038), tumor size (*P* = 0.044), pathology (*P* = 0.015), TNM stage (*P* = 0.001), PLR (*P* < 0.001), and LMR (*P* < 0.001). The PLR was significantly correlated with preoperative diabetes mellitus (*P* = 0.027), tumor size (*P* = 0.005), neutrophil count (*P* < 0.001), monocyte count (*P* = 0.034), NLR (*P* < 0.001), and LMR (*P* < 0.001). The LMR was significantly correlated with gender (*P* = 0.001), age (*P* = 0.007), smoking history (*P* < 0.001), preoperative emphysema (*P* = 0.013), preoperative FEV1/FVC (*P* = 0.004), tumor size (*P* = 0.037), TNM stage (*P* = 0.039), neutrophil count (*P* = 0.006), NLR (*P* < 0.001), and PLR (*P* < 0.001). It is notable that these three ratios were significantly associated with each other, which indicates that they may interact with each other. More information is shown in Table 2.

**Table 2:** Associations between the NLR, PLR, and LMR and clinicopathologic characteristics

	NLR			PLR			LMR		
Characteristics	< 2.12 (*n* = 141)	≥ 2.12 (*n* = 120)	*P*	< 92.9 (*n* = 127)	≥ 92.9 (*n* = 134)	*P*	< 4.57 (*n* = 131)	≥ 4.57 (*n* = 130)	*P*
Male, N (%)	67 (47.52)	77 (64.17)	**0.007**	63 (49.61)	81 (60.45)	0.078	86 (65.65)	58 (44.62)	**0.001**
Age, median (range), year	62 (38–77)	64 (41–81)	**0.017**	62 (38–77)	63 (39–81)	0.292	64 (40–81)	61 (38–77)	**0.007**
Smoking history, N (%)	54 (38.3)	69 (57.5)	**0.002**	54 (42.52)	69 (51.49)	0.147	76 (58.02)	47 (36.15)	**<0.001**
Preoperative comorbidity, N (%)									
Hypertension	35 (24.82)	44 (36.67)	**0.038**	35 (27.56)	44 (32.84)	0.354	45 (34.35)	34 (26.15)	0.15
Diabetes mellitus	13 (9.22)	13 (10.83)	0.664	18 (14.17)	8 (5.97)	**0.027**	12 (9.16)	14 (10.77)	0.664
COPD	23 (16.31)	16 (13.33)	0.501	17 (13.39)	22 (16.42)	0.492	24 (18.32)	15 (11.54)	0.124
CHD	7 (4.96)	5 (4.17)	0.759	7 (5.51)	5 (3.73)	0.492	7 (5.34)	5 (3.85)	0.564
Emphysema	20 (14.18)	23 (19.17)	0.28	16 (12.6)	27 (20.15)	0.1	29 (22.14)	14 (10.77)	**0.013**
Preoperative lung function									
FEV1, median (range), L	2.20 (0.76–4.2)	2.19 (0.92–4.55)	0.527	2.15 (0.76–4.2)	2.22 (0.92–4.55)	0.882	0.92(2.21–4.55)	2.16 (0.76–4.2)	0.606
FVC, median (range), L	2.96 (1.55–5.8)	3.13 (1.06–5.72)	0.921	2.98 (1.06–5.8)	3.05 (1.54–5.72)	0.466	3.17 (1.06–5.72)	2.92 (1.35–5.8)	0.34
FEV1/FVC, median (range), %	76.4 (38.91–90.4)	76 (36.8–96.49)	0.179	76.45 (36.8–96.49)	75.88 (40.54–89.77)	0.121	74.3 (36.8–92.92)	78.42 (38.91–96.49)	**0.004**
Tumor size, median (range), cm	2.2 (0.5–7)	2.55 (0.6–6.7)	**0.044**	2.2 (0.5–7)	2.6 (0.5–7)	**0.005**	2.5 (0.7–7)	2.25 (0.5–7)	**0.037**
Pathology (AC), N (%)	110 (78.01)	80 (66.67)	**0.015**	92 (72.44)	98 (73.13)	0.701	90 (68.7)	100 (76.92)	0.158
Resection (lobectomy), N (%)	98 (69.5)	92 (76.67)	0.195	86 (67.72)	104 (77.61)	0.073	96 (73.28)	94 (72.31)	0.86
TNM stage (I–II), N (%)	126 (89.36)	88 (73.33)	**0.001**	110 (86.61)	104 (77.61)	0.059	101 (77.1)	113 (86.92)	**0.039**
Neutrophil count, median (range), 10*9/L	2.97 (1.32–5.9)	4.20 (2.36–12.68)	**<0.001**	3.31 (1.32–8.26)	3.81 (1.46–12.68)	**<0.001**	3.8 (1.52–12.68)	3.36 (1.32–10.9)	**0.006**
Platelet count, median (range), 10*9/L	163 (70–337)	163.5 (73–391)	0.619	140 (70–337)	196.5 (73–391)	**<0.001**	160 (73–391)	170 (70–368)	0.342
Monocyte count, median (range), 10*9/L	0.37 (0.08–0.96)	0.39 (0.1–1.26)	0.072	0.38 (0.14–0.86)	0.38 (0.08–1.26)	**0.034**	0.45 (0.26–1.26)	0.32 (0.08–0.67)	**<0.001**
Lymphocyte count, median (range), 10*9/L	1.96 (0.98–4.32)	1.455 (0.56–2.53)	**<0.001**	1.95 (1.03–4.32)	1.5 (0.56–3.02)	**<0.001**	1.48 (0.56–2.8)	1.97 (0.58–4.32)	**<0.001**
NLR, median (range), %	1.6 (0.7–2.11)	2.88 (2.12–20.45)	**<0.001**	1.66 (0.7–5.1)	2.5 (0.91–20.45)	**<0.001**	2.44 (1.01–20.45)	1.65 (0.7–8.53)	**<0.001**
PLR, median (range), %	84.25 (35.9–167.28)	119.89 (39.48–323.33)	**<0.001**	76.72 (35.9–92.83)	124.36 (92.92–323.33)	**<0.001**	111.25 (37.17–323.33)	86.09 (35.9–266.67)	**<0.001**
LMR, median (range), %	5.49 (1.81–15.57)	3.71 (0.89–10.73)	**<0.001**	5.49 (2.6–15.57)	3.95 (0.89–12.25)	**<0.001**	3.46 (0.89–4.56)	6 (4.58–15.57)	**<0.001**

Bold text indicates significance. AC, adenocarcinoma; CHD, coronary heart disease; COPD, chronic obstructive pulmonary disease; FEV1, forced expiratory volume in one second; FVC, forced vital capacity; LMR, lymphocyte‐to‐monocyte ratio; NLR, neutrophil‐to‐lymphocyte ratio; PLR, platelet‐to‐lymphocyte; SC squamous carcinoma; SD, standard deviation; SD, standard deviation; TNM, tumor node metastasis.

### Associations between the IRB score and clinicopathologic characteristics

The IRB score was significantly correlated with the gender (*P* = 0.002), age (*P* = 0.024), smoking history (*P* = 0.001), preoperative hypertension (*P* = 0.018), preoperative emphysema (*P* = 0.042), preoperative FEV1/FVC (*P* = 0.005), tumor size (*P* = 0.004), extent of resection (*P* = 0.046), TNM stage (*P* = 0.009), neutrophil count (*P* < 0.001), platelet count (*P* = 0.007), monocyte count (*P* < 0.001), lymphocyte count (*P* < 0.001), NLR (*P* < 0.001), PLR (*P* < 0.001), and LMR (*P* < 0.001). Specific data is shown in Table 3.

**Table 3:** Associations between the IRB score and clinicopathological characteristics

	IRB score		
Characteristics	< 2 (*n* = 128)	≥ 2 (*n* = 133)	*P*
Male, N (%)	58 (45.31)	86 (64.66)	**0.002**
Age, median (range), year	62 (38–77)	64 (41–81)	**0.024**
Smoking history, N	47 (36.72)	76 (57.14)	**0.001**
Preoperative comorbidity, N (%)			
Hypertension	30 (23.44)	49 (36.84)	**0.018**
Diabetes mellitus	13 (10.16)	13 (9.77)	0.918
COPD	17 (13.28)	22 (16.54)	0.46
CHD	6 (4.69)	6 (4.51)	0.946
Emphysema	15 (11.72)	28 (21.05)	**0.042**
Preoperative lung function			
FEV1, median (range), L	2.22 (0.76–4.2)	2.18 (0.92–4.55)	0.382
FVC, median (range), L	2.97 (1.35–5.8)	3.1 (1.06–5.72)	0.588
FEV1/FVC, %	78.05 (38.91–90.4)	75 (36.8–96.49)	**0.005**
Tumor size, median (range), cm	2.2 (0.5–7)	2.6 (0.6–7)	**0.004**
Pathology (AC), N (%)	98 (76.56)	92 (69.17)	0.095
Resection (lobectomy), n (%)	86 (67.19)	104 (78.20)	**0.046**
TNM stage (I–II), N (%)	113 (88.28)	101 (75.94)	**0.009**
Neutrophil count, median (range), 10*9/L	3.17 (1.32–8.26)	3.94 (1.73–12.68)	**<0.001**
Platelet count, median (range), 10*9/L	156.5 (70–337)	181 (73–391)	**0.007**
Monocyte count, median (range), 10*9/L	0.36 (0.08–0.86)	0.42 (0.1–1.26)	**<0.001**
Lymphocyte count, median (range), 10*9/L	2 (0.98–4.32)	1.46 (0.56–2.55)	**<0.001**
NLR, median (range), %	1.61 (0.7–5.1)	2.78 (1.17–20.45)	**<0.001**
PLR, median (range), %	80.87 (35.9–167.28)	122.41 (39.48–323.33)	**<0.001**
LMR, median (range), %	5.69 (2.6–15.57)	3.53 (0.89–10.73)	**<0.001**

AC, adenocarcinoma; CHD, coronary heart disease; COPD, chronic obstructive pulmonary disease; FEV1, forced expiratory volume in one second; FVC, forced vital capacity; IRB, inflammatory response biomarker; LMR, lymphocyte‐to‐monocyte ratio; NLR, neutrophil‐to‐lymphocyte ratio; PLR, platelet‐to‐lymphocyte; SC squamous carcinoma; SD, standard deviation; SD, standard deviation; TNM, tumor node metastasis; bold text indicates significance.

### Postoperative overall survival (OS) and disease‐free survival (DFS) based on IRB score

**Figure 2:**
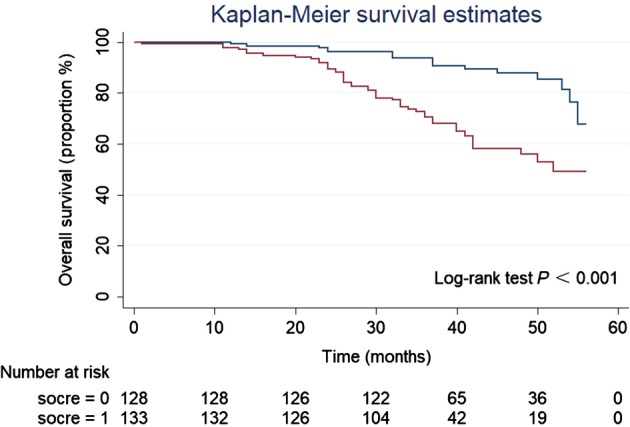
Kaplan–Meier survival curves showing the correlation between the inflammatory response biomarker (IRB) score and overall survival in 261 non‐small cell lung cancer patients. (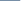) IRB score < 2, and (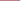) IRB score ≥ 2.

**Figure 3:**
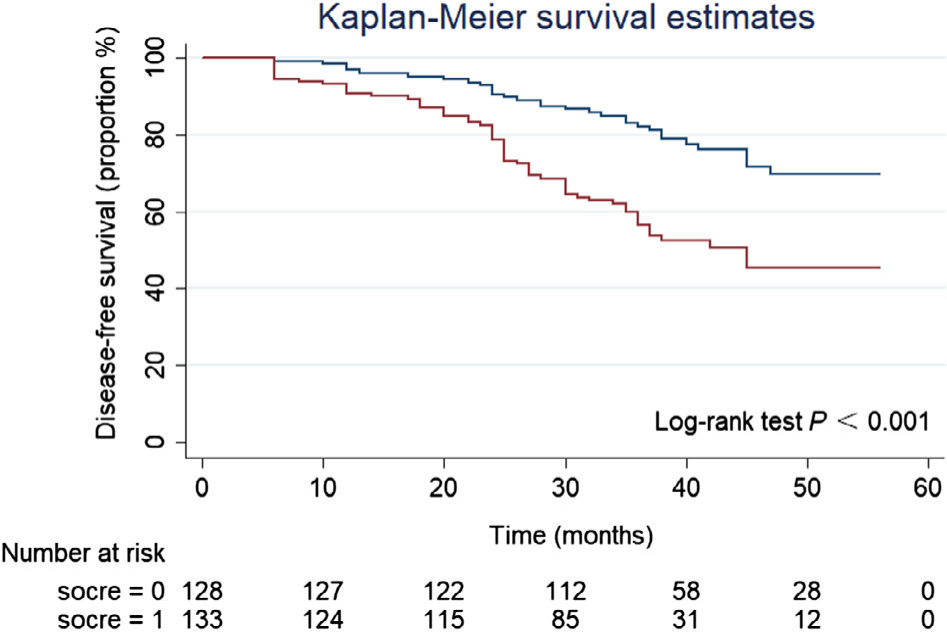
Kaplan–Meier survival curves showing the correlation between the inflammatory response biomarker (IRB) score and disease‐free survival in 261 non‐small cell lung cancer patients. (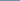) IRB score < 2, and (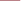) IRB score ≥ 2.

During the follow‐up period, the mean survival time of the 65 patients who died was 30.74 months, while the mean survival time of the 196 patients who were still alive was 41.78 months. The mean time to recurrence in 77 patients who experienced recurrence was 25.34 months compared to 41 months in 184 non‐recurrent patients. Patients with an IRB score ≤ 1 had significantly improved OS (*P* < 0.001) (Fig 2) and DFS (*P* < 0.001) (Fig 3) compared to patients with an IRB score ≥ 2.

### Prognostic factors of OS and DFS

Univariate analyses of OS revealed that the male gender (hazard ratio [HR] 1.689, 95% confidence interval [CI] 1.019–2.829; *P* = 0.042), age (HR 1.025, 95% CI 0.995–1.056; *P* = 0.098), smoking history (HR 2.272, 95% CI 1.370–3.768; *P* = 0.001), preoperative emphysema (HR 2.187, 95% CI 1.251–3.822; *P* = 0.006), larger tumors (HR 1.218, 95% CI 1.053–1.409; *P* = 0.008), high TNM stage (HR 1.926, 95% CI 1.236–3.338; *P* < 0.001), NLR ≥ 2.12 (HR 2.272, 95% CI 1.370–3.768; *P* = 0.005), PLR ≥ 92.9 (HR 2.064, 95% CI 1.234–3.451; *P* = 0.006), LMR < 4.57 (HR 3.067, 95% CI 1.801–5.223; *P* < 0.001), and IRB score ≥ 2 (HR 3.579, 95% CI 2.052–6.241; *P* < 0.001) were potential risk factors for poor OS. In multivariate analyses, high TNM stage (HR 2.721, 95% CI 1.597–4.989; *P* < 0.001) and IRB score ≥ 2 (HR 2.696, 95% CI 1.506–4.826; *P* = 0.001) were independently associated with poorer OS (Table 4).

**Table 4:** Univariate and multivariate Cox regression analyses of overall survival

	Univariate analysis			Multivariate analysis		
			Model 1		Model 2	
Characteristics	HR (95% CI)	*P*	HR (95% CI)	*P*	HR (95% CI)	*P*
Male	1.698 (1.019–2.829)	**0.042**	0.594 (0.189–1.861)	0.371	0.605 (0.202–1.812)	0.369
Age	1.025 (0.995–1.056)	0.098	1.024 (0.994–1.056)	0.117	1.018 (0.989–1.049)	0.221
Smoking history	2.272 (1.370–3.768)	**0.001**	3.021 (0.969–9.414)	0.057	2.738 (0.929–8.073)	0.068
Hypertension	1.004(0.593–1.700)	0.988				
Diabetes mellitus	0.862 (0.372–1.998)	0.862				
COPD	1.157 (0.588–2.274)	0.673				
CHD	1.100 (0.345–3.507)	0.873				
Emphysema	2.187 (1.251–3.822)	**0.006**	1.266 (0.694–2.307)	0.442	1.384 (0.764–2.505)	0.284
Preoperative lung function						
FEV1	0.873 (0.608–1.255)	0.463				
FVC	1.012 (0.746–1.374)	0.939				
FEV1/FVC	0.459 (0.075–2.807)	0.399				
Tumor size	1.218(1.053–1.409)	**0.008**	1.065 (0.911–1.245)	0.428	1.063 (0.911–1.239)	0.438
Pathology	0.914 (0.567–1.476)	0.714				
Lobectomy	1.275 (0.715–2.272)	0.410				
TNM stage	1.926 (1.482–2.503)	**< 0.001**	2.787 (1.604–4.843)	**<0.001**	2.721 (1.597–4.637)	**< 0.001**
NLR ≥ 2.12	2.031 (1.236–3.338)	**0.005**	0.849 (0.458–1.575)	0.604		
PLR ≥ 92.9	2.064 (1.234–3.451)	**0.006**	1.417 (0.777–2.584)	0.256		
LMR < 4.57	3.067 (1.801–5.223)	**< 0.001**	1.099 (0.629–1.919)	0.741		
IRB score ≥ 2	3.579 (2.052–6.241)	**< 0.001**			2.696 (1.506–4.826)	**0.001**

† Model 1 included the neutrophil‐to‐lymphocyte ratio (NLR), PLR, platelet‐to‐lymphocyte ratio (PLR), and lymphocyte‐to‐monocyte ratio (LMR) into multivariate analysis; Model 2 included the inflammatory response biomarker (IRB) score into multivariate analysis. Bold text indicates significance. AC, adenocarcinoma; CHD, coronary heart disease; CI, confidence interval; COPD, chronic obstructive pulmonary disease; FEV1, forced expiratory volume in one second; FVC, forced vital capacity; HR, hazard ratio; SC, squamous carcinoma; TNM, tumor node metastasis.

Univariate analyses of DFS showed that gender (HR 1.622, 95% CI 1.015–2.590; *P* = 0.043), smoking history (HR 2.001, 95% CI 1.265–3.165; *P* = 0.003), preoperative emphysema (HR 1.890, 95% CI 1.125–3.176; *P* = 0.016), FEV1/FVC (HR 0.234, 95% CI 0.046–1.196; *P* = 0.081), larger tumors (HR 1.230, 95% CI 1.077–1.406; *P* = 0.002), high TNM stage (HR 2.772, 95% CI 2.196–3.500; *P* < 0.001), NLR ≥ 2.12 (HR 3.082, 95% CI 1.901–4.996; *P* < 0.001), PLR ≥ 92.9 (HR 2.451, 95% CI 1.512–3.973; *P* < 0.001), LMR < 4.57 (HR 3.509, 95% CI 1.025–12.002; *P* = 0.046), and IRB score ≥ 2 (HR 4.258, 95% CI 2.504–7.241; *P* < 0.001) were potentially correlated with DFS. Multivariate analyses indicated that smoking history (HR 2.953, 95% CI 1.086–8.026; *P* = 0.034), TNM stage III (HR 3.108, 95% CI 1.911–5.056; *P* < 0.001), and IRB score ≥ 2 (HR 2.316, 95% CI 1.389–3.861; *P* = 0.001) were independently associated with poor DFS (Table 5).

**Table 5:** Univariate and multivariate Cox regression analyses of disease‐free survival

	Univariate analysis			Multivariate analysis		
			Model 1		Model 2	
Characteristics	HR (95% CI)	*P*	HR (95% CI)	*P*	HR (95% CI)	*P*
Male	1.622 (1.015–2.590)	**0.043**	0.388 (0.138–1.090)	0.072	0.402 (0.147–1.101)	0.076
Age	1.013 (0.986–1.041)	0.354				
Smoking history	2.001 (1.265–3.165)	**0.003**	2.938 (1.042–8.284)	**0.042**	2.953 (1.086–8.026)	**0.034**
Hypertension	1.125 (0.698–1.813)	0.629				
Diabetes mellitus	1.043 (0.501–2.169)	0.910				
COPD	1.221 (0.673–2.217)	0.512				
CHD	0.857 (0.270–2.719)	0.793				
Emphysema	1.890 (1.125–3.176)	**0.016**	1.598 (0.894–2.856)	0.113	1.667 (0.942–2.949)	0.079
Preoperative lung function						
FEV1	0.973 (0.696–1.360)	0.873				
FVC	1.055 (0.796–1.399)	0.710				
FEV1/FVC	0.234 (0.046–1.196)	0.081	1.239 (0.205–7.504)	0.815	1.324 (0.220–7.948)	0.759
Tumor size	1.230 (1.077–1.406)	**0.002**	1.132 (0.984–1.302)	0.082	1.131 (0.985–1.298)	0.081
Pathology	1.157 (0.753–1.778)	0.505				
Lobectomy	1.072 (0.644–1.786)	0.788				
TNM stage	2.772 (2.196–3.500)	**< 0.001**	3.100 (1.871–5.135)	**< 0.001**	3.108 (1.911–5.056)	**< 0.001**
NLR ≥ 2 .12	3.082 (1.901–4.996)	**< 0.001**	0.989 (0.561–1.744)	0.969		
PLR ≥ 92.9	2.451 (1.512–3.973)	**< 0.001**	1.180 (0.678–2.052)	0.559		
LMR < 4.57	3.509 (1.025–12.002)	**0.046**	1.073 (0.658–1.748)	0.777		
IRB score ≥ 2	4.258 (2.504–7.241)	**< 0.001**			2.316 (1.389–3.861)	**0.001**

† Model 1 included the neutrophil‐to‐lymphocyte ratio (NLR), PLR, platelet‐to‐lymphocyte ratio (PLR), and lymphocyte‐to‐monocyte ratio (LMR) into multivariate analysis; Model 2 included the inflammatory response biomarker (IRB) score into multivariate analysis. Bold text indicates significance. AC, adenocarcinoma; CHD, coronary heart disease; CI, confidence interval; COPD, chronic obstructive pulmonary disease; FEV1, forced expiratory volume in one second; FVC, forced vital capacity; HR, hazard ratio; SC, squamous carcinoma; TNM, tumor node metastasis.

## Discussion

In our study, we evaluated associations among NLR, PLR, LMR, and IRB scores with OS and DFS in NSCLC patients who underwent thoracoscopic radical resection. The main finding was that the IRB score was an independent prognostic factor in NSCLC patients and was superior to NLR, PLR, and LMR alone for predicting prognosis.

Over the past decade, numerous studies have shown a correlation between the SIR and several types of solid cancers, including lung cancer.^16^, ^17^, ^18^, ^19^, ^20^ Previous research demonstrated that the SIR could influence tumor progression by regulating the invasive and metastatic potential of lung cancer cells^21^ there are also many biomarkers that could represent systemic inflammation well. Unfortunately, some of these are not clinically accessible or are only applied as research tools, such as the modified Glasgow prognostic score (mGPS, a composite score derived from albumin levels and CRP).^22^ A blood test is easy to obtain and is inexpensive, and almost every patient undergoes this test before surgery. Therefore, it would be very helpful for clinicians to develop and adjust treatment strategies if lung cancer prognosis could be predicted through a routine blood examination.

The association between blood cell counts or their ratios with lung cancer prognosis has been explored.^23^, ^24^, ^25^ Yin *et al.* conducted a meta‐analysis including 2734 patients from 14 studies and reported that a high NLR was a predictor of poor OS in lung cancer (HR 1.192, 95% CI 1.061–1.399; *P*_heterogeneity_ = 0.003).^23^ Zhang *et al.* also conducted a meta‐analysis of 2889 patients from 12 studies and reported that NSCLC patients with an elevated PLR were more likely to have shorter OS after therapy (HR 1.492, 95% CI 1.231–1.807; *P* < 0.001).^24^ Furthermore, in their meta‐analysis including 3954 patients from eight studies, Li *et al.* demonstrated that a low LMR was significantly associated with poorer OS (HR 1.651, 95% CI 1.306–2.086, *P* < 0.001) and progression‐free survival (PFS, HR 1.431, 95% CI 1.294–1.582, *P* < 0.001).^25^ However, our results indicated that none of the three ratios were independent prognostic factors for OS of NSCLC, thus a larger sample is required to verify their value for predicting NSCLC prognosis.

Although the mechanisms by which the SIR affects lung cancer prognosis are not yet clear, some significant progress has been reported. Tumor‐associated neutrophils (TANs), which are derived from peripheral neutrophils, are considered key mediators in tumor progression because they can accelerate tumor growth, stimulate angiogenesis, cause genetic instability, and improve the invasiveness of tumor cells.^26^ Tumor‐associated macrophages (TAMs), which are derived from circulating monocytic precursors, play a key role in the inflammatory microenvironment of tumor progression.^26^ TAMs can produce angiogenic and growth factors, as well as the protease enzyme, which promote the degradation of extracellular matrixes and induce angiogenesis, accelerate tumor cell proliferation, and favor metastasis and invasion.^27^ Unlike neutrophils and monocytes, lymphocytes play an essential role in the regulation of host cell‐mediated immunity, which is helpful for destroying residual malignant cells and related micrometastases.^28^ Meanwhile, it is well known that tumor‐infiltrating lymphocytes (TILs) are correlated with improved clinical outcomes in cancers.^29^ Recent studies have reported that platelets are important for tumor angiogenesis.^30^, ^31^ The mechanism may be that platelets adhere to tumor vessels and release granules containing potent angiogenesis stimulators, such as the platelet‐derived endothelial cell growth factor.^30^, ^31^ All of the mechanisms we examined may explain why patients with a high NLR, a high PLR, or a low LMR have poor survival rates.

Most studies of the prognostic significance of SIR only focused on single factors and did not attempt to combine these biomarkers. Our study described a novel scoring system that utilized the combination of NLR, PLR, and LMR, the IRB score. The IRB was superior to the single ratios for predicting NSCLC prognosis, indicating that this measure may be more useful than these ratios alone.

Our study excluded patients who experienced a conversion from thoracoscopic surgery to thoracotomy. According to research by Oda *et al.*, thoracoscopic surgery could reduce blood loss, the duration of chest tube placement, postoperative hospital stay and CRP level, and improve five‐year OS and DFS compared to thoracotomy in early NSCLC patients.^32^ Their results indicated that the surgical method had a significant influence on peripheral blood inflammatory biomarkers and prognosis. Therefore, patients undergoing a change to thoracotomy were excluded from our study to decrease the bias caused by the surgical approach.

There were some limitations to our study. First, it was a retrospective study from a single center, thus the sample size was small. Second, the median follow‐up was 38 (range: 1–56) months; therefore we were not able to observe the predictive effect of the IRB score on long‐term prognosis. Third, we excluded patients who underwent another surgery within three months before thoracoscopic surgery because surgery can cause changes to the SIR. However, whether the interval of three months was sufficient is unclear. Four, the optimal cutoff values of the NLR, PLR, and LMR in our study were 2.12, 92.9, and 4.57, respectively. In previous studies, their critical values ranged from 2.5 to 5, 106 to 300, and 2.62 to 4.56, respectively;^23^, ^24^, ^25^ therefore, the critical values in the current study may only be appropriate to the population of our center. If physicians from other medical centers attempt to apply this prognostic scoring system, we suggest that they perform their own analysis to obtain the local cutoff values that are appropriate to the specific patient population. Five, we only enrolled the NLR, PLR, and LMR into our prognostic scoring system. There are additional systemic inflammatory biomarkers with high prognostic significance, such as the prognostic nutritional index and mGPS.^22^, ^33^ Unfortunately, they were not routinely available in our department. Future studies of the prognostic scoring system should include as many systemic inflammation indicators as possible. Finally, because of a lack of external data, our results could not be further validated.

In conclusion, in this study we developed a novel prognostic scoring system using the NLR, PLR, and LMR that we termed the IRB score, and demonstrated that an IRB score ≥ 2 was an independent prognostic factor for poor survival. Additional prospective multicenter studies are needed to confirm its prognostic significance in NSCLC.

## Disclosure

No authors report any conflict of interest.
